# Schizophrenia-associated dysbindin modulates axonal mitochondrial movement in cooperation with p150^glued^

**DOI:** 10.1186/s13041-020-00720-3

**Published:** 2021-01-18

**Authors:** Bo Kyoung Suh, Seol-Ae Lee, Cana Park, Yeongjun Suh, Soo Jeong Kim, Youngsik Woo, Truong Thi My Nhung, Su Been Lee, Dong Jin Mun, Bon Seong Goo, Hyun Sun Choi, So Jung Kim, Sang Ki Park

**Affiliations:** 1 Department of Life Sciences, Pohang University of Science and Technology, Pohang, Republic of Korea; 2 Weill Institute of Neurosciences, Department of Neurology, University of California, San Francisco, San Francisco, USA; 3 Department of Chemical Engineering, Pohang University of Science and Technology, Pohang, Republic of Korea

**Keywords:** Mitochondrial movement, Calcium homeostasis, Dysbindin, p150^glued^, Dynactin complex

## Abstract

Mitochondrial movement in neurons is finely regulated to meet the local demand for energy and calcium buffering. Elaborate transport machinery including motor complexes is required to deliver and localize mitochondria to appropriate positions. Defects in mitochondrial transport are associated with various neurological disorders without a detailed mechanistic information. In this study, we present evidence that dystrobrevin-binding protein 1 (dysbindin), a schizophrenia-associated factor, plays a critical role in axonal mitochondrial movement. We observed that mitochondrial movement was impaired in dysbindin knockout mouse neurons. Reduced mitochondrial motility caused by dysbindin deficiency decreased the density of mitochondria in the distal part of axons. Moreover, the transport and distribution of mitochondria were regulated by the association between dysbindin and p150^glued^. Furthermore, altered mitochondrial distribution in axons led to disrupted calcium dynamics, showing abnormal calcium influx in presynaptic terminals. These data collectively suggest that dysbindin forms a functional complex with p150^glued^ that regulates axonal mitochondrial transport, thereby affecting presynaptic calcium homeostasis.

## Introduction

Neurons harbor several hundred mitochondria along their projections because of their massive energy demand and calcium homeostasis. Proper distribution of mitochondria is achieved by bidirectional mitochondrial movement along microtubule-based tracks through collaborations of various motor proteins (such as dynein and kinesin) and motor adaptors [1, 2].

Dynactin is a multi-subunit protein complex required for dynein-mediated transport of cargos, including vesicles and organelles, along the microtubule. p150^glued^, also known as dynactin 1, is crucial for dynein motor activity while the other subunits maintain the structure of the complex [3, 4]. p150^glued^ recruits dynein to the plus ends of microtubules and membranous cargos, allowing the motor proteins to achieve long-distance and processive transport along the microtubules [5, 6]. Animal models with disrupted dynein-dynactin complex exhibit symptoms of neurodegenerative diseases, including motor neuron degeneration. Cytoskeleton-associated protein glycine-rich (CAP-Gly) domain of p150^glued^ is essential for the microtubule-binding affinity of p150^glued^ and mutations in this domain cause defects in spinal neurons [7]. Similarly, mutations in p150^glued^ have been reported in patients with neurodegenerative diseases, such as spinobulbar muscular atrophy (SBMA) and amyotrophic lateral sclerosis (ALS) [8].

Defects in the mitochondrial transport are also related to a considerable number of neurological disorders. Notably, the impaired axonal mitochondrial transport in Alzheimer’s disease and Parkinson’s disease are related to accumulations of amyloid β (Aβ) and the higher level of α-synuclein aggregation, respectively [9, 10]. Reduced mitochondrial density in soma and axon terminals were observed in patients with schizophrenia [11–13]. In addition, abnormal intracellular calcium homeostasis, regulated by mitochondria and endoplasmic reticulum (ER), is implicated in schizophrenia [14–17]. However, the precise mechanistic basis of the link has not been clearly elucidated.

Dystrobrevin-binding protein 1 (dysbindin) is a coiled-coil-containing protein that plays diverse roles in neuronal morphogenesis, synaptic vesicle biogenesis, exocytosis, and dendritic spinogenesis [18–22]. The association of dysbindin and schizophrenia was revealed by human postmortem studies of schizophrenic patients showing decreased expression levels of dysbindin in the brain and impaired cognitive function [23–25]. Dysbindin-knockout mice (*Sandy*) show abnormalities in dendritic morphogenesis, long-term potentiation, and synaptic transmission [26, 27], along with schizophrenia-like behavioral phenotypes [28–30]. Besides, some studies including a proteomics analysis of dysbindin suggested its potential association with motor complexes [31–33]. Given that dysbindin was detected in the vicinity of microtubules and outer membranes of mitochondria concurrently [31], we hypothesized dysbindin might play a role in mitochondrial movement along microtubules, which can provide mechanistic insight into schizophrenia-related synaptic functions.

In this study, we newly demonstrate that dysbindin functions as a regulator of neuronal mitochondrial motility and distribution, which is achieved by functional coordination of dynactin motor complex. We also show the significance of this process for the local calcium homeostasis in the neuron.

## Results

### Dysbindin regulates the mitochondrial movement and distribution in axons

**Fig. 1:**
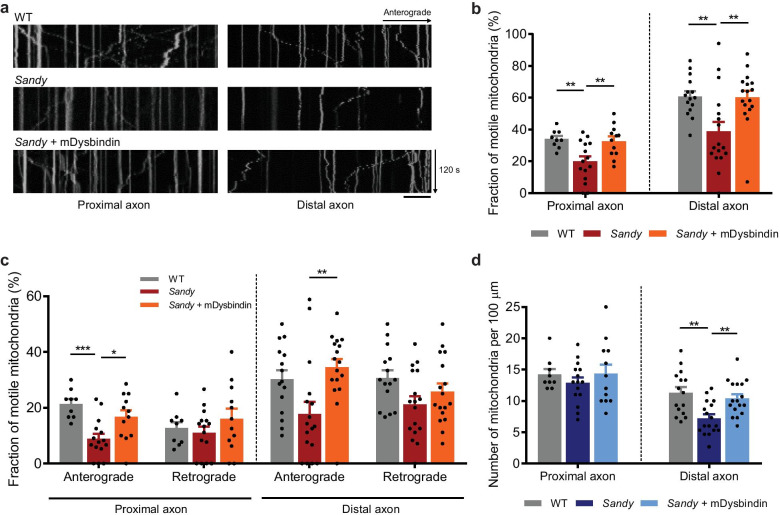
Dysbindin regulates axonal mitochondrial movement and density. **a** Representative kymographs of mitochondrial movement in DIV 10–12 primary cortical neurons of WT and *Sandy* mice. The movements of mitochondria in the proximal and distal axons are shown. A scale bar represents 20 μm. **b** Quantitative analyses of the motile fraction of mitochondria, and **c** anterograde and retrograde movements of mitochondria. **d** Quantification of mitochondrial density (the number of mitochondria per 100 μm) in the proximal and distal axons of DIV 10–12 primary cortical neurons (n = 9 axons for WT, n = 15 axons for *Sandy*, and n = 12 axons for *Sandy* + mDysbindin for the proximal axons, n = 15 axons for WT, n = 17 axons for *Sandy*, and n = 17 axons for *Sandy* + mDysbindin for the distal axons). All results are presented as the mean $\pm$ SEM. *p < 0.05, **p < 0.01, and ***p < 0.001 from one-way ANOVA with Bonferroni’s multiple comparison test

To identify the regulatory function of dysbindin in the movement of mitochondria, we measured axonal transport of mitochondria in primary cultured cortical neurons from wild-type (WT) and *Sandy* mouse embryos. Mitochondrial motility represented by the fraction of motile axonal mitochondria in *Sandy* neurons decreased significantly relative to that in WT neurons (Fig. 1a, b). The defects were effectively reversed by the overexpression of dysbindin, indicating that the defects in mitochondrial movement are specific to dysbindin deficiency. When the directionality of axonal movement of mitochondria was analyzed, the anterograde movement decreased significantly whereas the retrograde movement remained intact (Fig. 1c).

To determine whether the defects in mitochondrial motility are extended to the mitochondrial distribution in neurons, we examined mitochondrial density along the axon. We selected two axonal areas for time-lapse imaging: a 150 µm region proximal to the soma (proximal axon) and a 150 µm region from the axon tip, including a growth cone (distal axon). As a result, while the mitochondrial density in the proximal axons of the *Sandy* neurons was not altered, the distal axons in the *Sandy* neurons displayed a significant decrease in density that was effectively reversed by dysbindin overexpression (Fig. 1d). These results were recapitulated in the short hairpin ribonucleic acid (shRNA)-based dysbindin knockdown experiment. The knockdown effects were reversed by shRNA-resistant dysbindin co-expression, suggesting that the alterations in mitochondrial motility in the proximal axons and mitochondrial density in the distal axons were also specific to dysbindin depletion (Additional file 1: Fig. S1a-c). In agreement with the above results, dysbindin overexpression showed an increase in the fraction of motile mitochondria (Additional file 1: Fig. S1d-f). The effect of dysbindin on mitochondrial motility and density was mostly intact in dendrites (Additional file 1: Fig. S1g, h), collectively suggesting that dysbindin is an important regulator in axonal mitochondrial transport.

### Dysbindin interacts with p150^glued^ to regulate its microtubule association

**Fig. 2:**
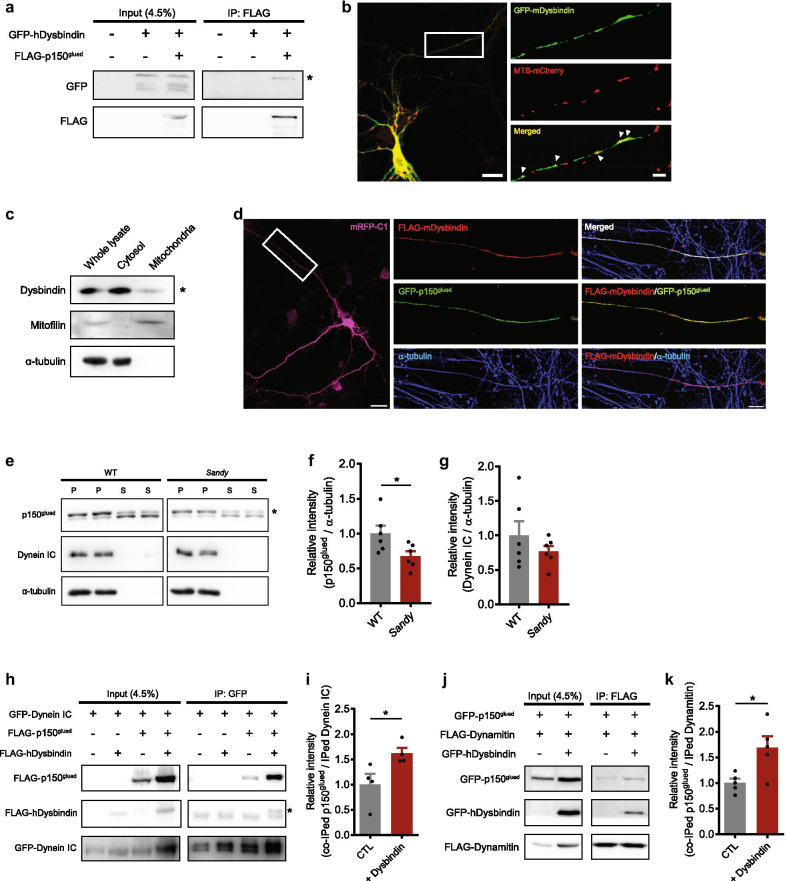
Dysbindin interacts with p150^glued^ affecting microtubule association and complex integrity. **a** Co-immunoprecipitation of dysbindin and p150^glued^. Lysates from HEK293 cells transfected with GFP-hDysbindin and FLAG-p150^glued^ constructs were immunoprecipitated with anti-FLAG antibody. Immunoprecipitates were analyzed by western blotting with anti-GFP and anti-FLAG antibodies. **b** Co-localization of dysbindin and mitochondria in the axon of DIV 10 primary cortical neurons. Neurons transfected with GFP-mDysbindin and MTS-mCherry were stained with anti-GFP antibody. Overlapped signals are indicated by arrow heads. Scale bars represent 20 μm (left) and 5 μm (right). **c** Endogenous dysbindin detected from the mitochondrial fraction of DIV 11 primary cultured neurons. Dysbindin was detected by western blotting with anti-dysbindin antibody. Mitofilin and α-tubulin were used as markers for mitochondrial and cytosolic fractions, respectively. **d** Co-localization of dysbindin and p150^glued^ along the microtubules in the axon of DIV 10 primary cortical neurons. Neurons transfected with mRFP-C1, FLAG-mDysbindin and GFP-p150^glued^ were stained with anti-FLAG (red) and anti-α-tubulin (blue) antibodies. Scale bars represent 20 μm (left) and 5 μm (right). **e** Microtubule co-sedimentation assay for p150^glued^ and dynein IC in mouse brain tissue. Polymerized tubulin in the pellet fraction (P) and non-polymerized tubulin in the supernatant (S) were subjected to western blotting with anti-p150^glued^ and anti-dynein IC antibodies. **f** Quantification of the protein level of p150^glued^ and **g** the protein level of dynein IC in the pellet fraction normalized by that of α-tubulin. **h** Co-immunoprecipitation of p150^glued^ and dynein IC upon dysbindin co-expression. Lysates from HEK293 cells transfected with indicated constructs were immunoprecipitated with anti-GFP antibody. Immunoprecipitates were analyzed by western blotting with anti-FLAG and anti-GFP antibodies. **i** Quantification of the protein level of co-immunoprecipitated p150^glued^ normalized by immunoprecipitated dynein IC. **j** Co-immunoprecipitation of p150^glued^ and dynamitin upon dysbindin co-expression. Lysates from HEK293 cells transfected with indicated constructs were immunoprecipitated with anti-FLAG antibody. Immunoprecipitates were analyzed by western blotting with anti-GFP and anti-FLAG antibodies. **k** Quantification of the protein level of co-immunoprecipitated p150^glued^ normalized by immunoprecipitated dynamitin. Asterisks indicate the protein of interest. All results are presented as the mean $\pm$ SEM. *p < 0.05, **p < 0.01, and ***p < 0.001 from Student’s *t* test

To identify the mechanistic link between dysbindin and motor complexes relevant to the mitochondrial transport, we first tested the physical association between dysbindin and p150^glued^, a strong candidate motor protein to interact with dysbindin [33]. Dysbindin was co-immunoprecipitated with p150^glued^ in human embryonic kidney (HEK) 293 cell lysates (Fig. 2a). We next investigated the subcellular localization of dysbindin to mitochondria by immunocytochemistry. We found that dysbindin signals overlapped with those of MTS-mCherry, a mitochondrial marker, in axons of primary cortical neurons (Fig. 2b). Moreover, a significant portion of endogenous dysbindin was detected in the mitochondrial fraction obtained from primary cultured neurons (Fig. 2c). Consistently, a substantial level of endogenous dysbindin protein was detected in the mitochondrial fraction of mouse brain lysates (Additional file 2: Fig. S2a). The level of p150^glued^ associated with mitochondria in the WT and *Sandy* mice brains were not different significantly (Additional file 2: Fig. S2b).

In the immunocytochemical analysis with primary cortical neurons, the dysbindin signal was co-localized with p150^glued^ along the axonal microtubule (Fig. 2d), supporting the notion that dysbindin is a microtubular protein participating in the cargo transport. Based on the potential involvement of dysbindin in microtubule-related processes [34, 35], we tested whether the association of p150^glued^ with microtubules is affected by dysbindin. We performed a microtubule co-sedimentation assay using mouse brain lysates and observed a decrease in co-sedimentation of p150^glued^ and microtubules in *Sandy* mouse brain tissue compared to that of WT (Fig. 2e, f). The co-sedimentation of dynein intermediate chain (dynein IC), which directly binds to p150^glued^, was not affected (Fig. 2g).

Next, to test whether dysbindin affects the integrity of dynein-dynactin motor complex, we examined the interaction between p150^glued^ and dynein IC. We observed a significant increase in the co-precipitation of p150^glued^ and dynein IC upon dysbindin overexpression (Fig. 2h, i). Besides, p150^glued^ co-immunoprecipitated with dynamitin (dynactin 2) significantly increased upon dysbindin overexpression (Fig. 2j, k). When dysbindin was knocked down, endogenous dynamitin co-immunoprecipitated with p150^glued^ was also reduced (Additional file 2: Fig. S2c-f). These results indicate that dysbindin participates in the regulation of motor complex integrity as well as the interaction between individual dynactin subunits.

### Dysbindin regulates axonal mitochondrial motility in cooperation with p150^glued^

**Fig. 3:**
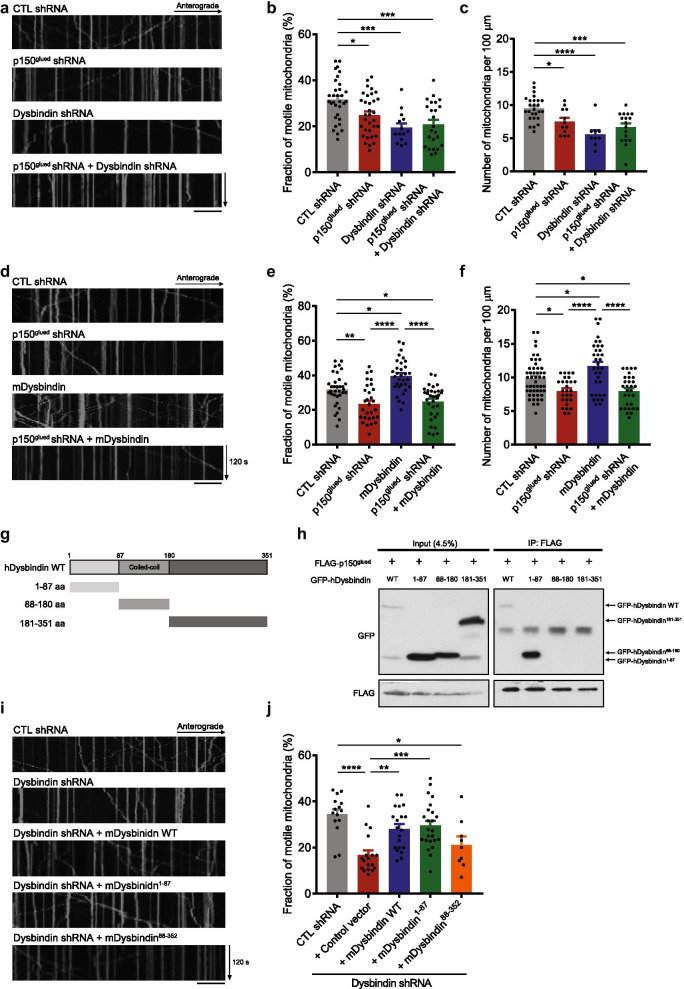
Dysbindin regulates axonal mitochondrial motility in cooperation with p150^glued^. **a** Representative kymographs of mitochondrial movement in the proximal axons of DIV 10–12 primary cortical neurons transfected as indicated. **b** Quantitative analysis of the motile fraction of mitochondria in the proximal axons (n = 32 axons for control (CTL) shRNA, n = 33 axons for p150^glued^ shRNA, n = 14 axons for Dysbindin shRNA, and n = 24 axons for p150^glued^ shRNA + Dysbindin shRNA). **c** Quantification of mitochondrial density (the number of mitochondria per 100 μm) in the distal axons of DIV 10–12 primary cortical neurons (n = 26 axons for CTL shRNA, n = 12 axons for p150^glued^ shRNA, n = 9 axons for Dysbindin shRNA, and n = 17 axons for p150^glued^ shRNA + Dysbindin shRNA). **d** Representative kymographs of mitochondrial movement in the proximal axons of DIV 10–12 primary cortical neurons transfected as indicated. **e** Quantitative analysis of the motile fraction of mitochondria in the proximal axons (n = 34 axons for CTL shRNA, n = 30 axons for p150^glued^ shRNA, n = 32 axons for mDysbindin, and n = 33 axons for p150^glued^ shRNA + mDysbindin). **f** Quantification of mitochondrial density (the number of mitochondria per 100 μm) in the distal axons of DIV 10–12 primary cortical neurons (n = 49 axons for CTL shRNA, n = 27 axons for p150^glued^ shRNA, n = 34 axons for mDysbindin, and n = 30 axons for p150^glued^ shRNA + mDysbindin). **g** Schematic diagrams of deletion mutants of dysbindin protein used for the co-immunoprecipitation analysis. aa, amino acids. **h** Co-immunoprecipitation of p150^glued^ with dysbindin fragments. Lysates from HEK293 cells transfected with FLAG-p150^glued^ and indicated GFP-hDysbindin fragments were immunoprecipitated with anti-FLAG antibody. Immunoprecipitates were analyzed by western blotting with anti-GFP and anti-FLAG antibodies. **i** Representative kymographs of mitochondrial movement in the proximal axons of DIV 10–12 primary cortical neurons transfected as indicated. **j** Quantitative analysis of the motile fraction of mitochondria in the proximal axons (n = 15 axons for CTL shRNA, n = 18 axons for Dysbindin shRNA + Control vector (pFLAG-CMV2), n = 20 axons for Dysbindin shRNA + mDysbindin WT, n = 24 axons for Dysbindin shRNA + mDysbindin 1–87 aa, and n = 9 axons for Dysbindin shRNA + mDysbindin 88–352 aa). Scale bars represent 20 μm. All results are presented as the mean $\pm$ SEM. *p < 0.05, **p < 0.01, and ***p < 0.001 from one-way ANOVA with Bonferroni’s multiple comparison test

To identify the functional relationship between dysbindin and p150^glued^ in mitochondrial transport, we analyzed whether dysbindin-mediated mitochondrial movement and distribution are altered by p150^glued^ depletion. In p150^glued^ knockdown, mitochondrial motility in the proximal axons was significantly reduced (Fig. 3a, b) and the density of mitochondria in the distal axons was also significantly reduced (Fig. 3c), as seen in the dysbindin depletion conditions. Moreover, additional impairment of mitochondrial motility in the proximal axons (Fig. 3b) or mitochondrial density in the distal axons (Fig. 3c) was not observed following the concomitant knockdown of p150^glued^ and dysbindin. Likewise, dysbindin overexpression did not restore the decrease in mitochondrial movement in the proximal axons or mitochondrial density in the distal axons caused by p150^glued^ knockdown (Fig. 3d–f). The results from mitochondrial density in the proximal axons and mitochondrial motility in the distal axons are shown in ‘Additional file 3: Fig. S3′. These data collectively indicate that dysbindin and p150^glued^ cooperate to regulate mitochondrial transport in the same motor machinery.

To determine a functional dysbindin domain involved in association with p150^glued^, we generated deletion mutants of dysbindin on the basis of domain predictions. Mostly, dysbindin interacts with binding partners via its coiled-coil structure, thus, we divided dysbindin protein into three parts, with the coiled-coil domain in the middle. The N-terminal region (1–87 aa) lies from the first amino acid to the beginning of the coiled-coil domain, the middle region (88–180 aa) covers the coiled-coil domain and a leucine zipper motif, and the C-terminal region (181–351 aa) contains a dysbindin domain and a PEST domain (Fig. 3g) [36–38]. In co-immunoprecipitation analysis using these dysbindin fragments, only the N-terminal region of dysbindin was co-immunoprecipitated with p150^glued^ (Fig. 3h). Consistently, the N-terminal region of dysbindin restored the mitochondrial movement impaired upon dysbindin knockdown, whereas it was not the case for dysbindin lacking the N-terminal region (Fig. 3i, j). These results suggest that the physical interaction between dysbindin and p150^glued^ is important for regulating mitochondrial transport.

### Dysbindin controls calcium homeostasis in presynaptic terminals

**Fig. 4:**
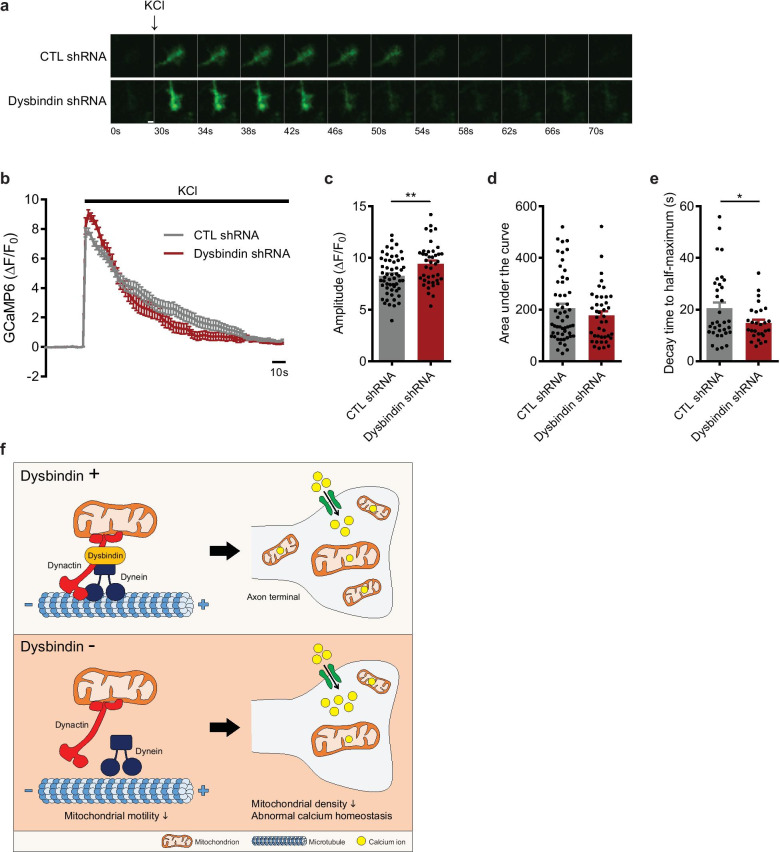
Dysbindin mediates calcium homeostasis in presynaptic terminals. **a** Representative time-lapse images of calcium responses in the axon tip of DIV 10–12 primary cortical neurons transfected with the indicated shRNA. A scale bar represents 2 μm. **b** Calcium response graph obtained after 50 mM KCl stimulation. **c** Statistically analyzed peak amplitudes, **d** the area under the curves, and **e** the decay time to half-maximum of the calcium response graph (n = 55 for CTL shRNA and n = 41 for Dysbindin shRNA). **f** A schematic model of the function of dysbindin in regulating mitochondrial movement. The association of dysbindin to dynactin complex facilitates the mitochondrial motility and thereby affects calcium dynamics in the axon tip. All results are presented as the mean $\pm$ SEM. *p < 0.05, **p < 0.01, and ***p < 0.001 from Student’s *t*-test

In the presynaptic terminal of axons, mitochondria regulate local calcium homeostasis [39, 40]. Therefore, we tested if the attenuated motility of mitochondria and the altered distribution of mitochondria in the distal axons result in altered local calcium dynamics. We performed time-lapse live-cell imaging using cyto-GCaMP6 in the axon tip of primary cultured cortical neurons after dysbindin knockdown (Fig. 4a, b). Potassium chloride (KCl)-stimulated axon terminal calcium influx increased in dysbindin-deficient neurons (Fig. 4c), whereas the amount of accumulated calcium did not change (Fig. 4d). Moreover, the decay time to half-maximum decreased in dysbindin-deficient neurons, showing faster off rates compared to the control neurons (Fig. 4e). These results were recapitulated in the comparison of calcium dynamics between WT and *Sandy* neurons, although the difference of the peak amplitudes between the two groups did not achieve statistical significance (Additional file 4: Fig. S4a–d). Increased calcium influx shown in the axon tip of dysbindin-deficient neurons was also reproduced in presynaptic boutons of dysbindin-deficient neurons expressing vGlut1-GCaMP5G, a presynaptic calcium sensor (Additional file 4: Fig. S4e–g). Collectively, it is likely that impaired presynaptic calcium buffering is caused by the reduced number of mitochondria resulting from dysbindin deficiency, further highlighting the critical role of dysbindin in synaptic activity.

## Discussion

In this study, we demonstrate that dysbindin regulates axonal mitochondrial movement in cooperation with dynactin complex. Dysbindin deficiency causes defects in mitochondrial transport along the axon and results in reduced mitochondrial density in the distal axons. Finally, it affects the local calcium homeostasis in axon terminals, resulting in abnormal calcium influx (Fig. 4f).

We proposed a novel functional link between dysbindin and the microtubule motor complex by multiple experimental approaches. Indeed, previous EM-based study has shown the existence of dysbindin in the vicinity of microtubule structure [31], hinting at their physical and functional association. Extending this observation, we demonstrated that the association of p150^glued^ with microtubules was significantly decreased in the *Sandy* mice. As p150^glued^ is required to recruit dynein motor to microtubules for the efficient initiation of transport and its processivity [5, 6], our results indicate that dysbindin plays a role as a motor adaptor, recruiting dynein-dynactin motor complex to microtubules in axons. Given that p150^glued^ in mitochondrial fraction was largely not changed in *Sandy* mice (Additional file 2: Fig. S2b), the function of dysbindin seems to be specific for the association of dynein-dynactin complex to microtubules but irrelevant to its association to cargos. In this regard, dysbindin may function as a novel adaptor or scaffolding protein, modulating the recruitment of dynein-dynactin complex to the microtubule and subsequently determining the efficiency of organelle and vesicle transports along the axon.

Dysbindin is a component of biogenesis of lysosome-related organelle complex 1 (BLOC-1) and a large number of studies on dysbindin have focused on its function as a constituent of BLOC-1 complex [41]. In particular, BLOC-1 and dysbindin are involved in transporting or positioning multiple targets in neurons. Dysbindin functions in vesicle transport mediated by BLOC-1 and adaptor related protein complex‐3 (AP-3) for sorting cargos from cell bodies to nerve terminals [42]. Specific subunits of BLOC-1, such as snapin, regulate lysosome trafficking along the microtubule by recruiting motor machinery to lysosomes [43]. Therefore, it would be interesting to see whether dysbindin and p150^glued^ cooperate to transport other cargos beyond mitochondria, such as lysosomes and synaptic vesicles, functioning as a general factor for many more cargos.

Dysbindin-deficient neurons showed an excessive calcium influx in the presynaptic axon tip (Fig. 4c). Recent reports support the notion that presynaptic calcium buffering is affected by the presence of mitochondria along with the attenuation of presynaptic calcium transients in response to action potentials and downregulation of synaptic vesicle release [44, 45]. Indeed, we demonstrated that dysbindin deficiency leads to a decreased local mitochondrial density in the axon tip (Fig. 1d), which likely to underlie the exaggerated local calcium increase. Lines of evidence have shown that dysbindin-deficient mice show decreased glutamate release [46, 47] and GABAergic transmission [48, 49] in the prefrontal cortex, which all are interlinked with presynaptic calcium transients. In addition, this impaired neurotransmission has been implicated in the etiology of schizophrenia [29, 50, 51]. Further work is required to identify whether the dysbindin-mediated regulation of calcium homeostasis is on the same line with previously known presynaptic functions of dysbindin mediating the trafficking and priming of synaptic vesicles.

Altered mitochondrial distribution in neurons is implicated in the pathogenesis of multiple psychiatric conditions, including schizophrenia. Patients with schizophrenia show defects in the distribution of axonal mitochondria in their caudate nucleus and putamen, as well as a decreased mitochondrial density in the axon terminal region in their anterior cingulate cortex [12, 13]. In this regard, the potential role of dysbindin-p150^glued^ complex in the movement and distribution of mitochondria may explain one of the pathological mechanisms of schizophrenia. Previously, it was reported that dysbindin forms a functional complex with disrupted-in-schizophrenia 1 (DISC1), another schizophrenia susceptibility factor. Their physical interaction is important for stabilizing dysbindin and regulating neurite outgrowth [38]. In addition, DISC1 has been reported to regulate axonal mitochondrial movement by anchoring mitochondria with syntaphilin [52, 53]. Therefore, it would be of immediate interest to investigate potential communications between dysbindin-containing motor complex and DISC1-containing mitochondrial anchoring machinery in the proper axonal mitochondrial positioning and related neurological disease conditions.

## Methods

### Animals

*Sandy* (*sdy*−/−) mice on DBA/2J background were obtained from the Jackson Laboratory (Bar Harbor, ME) [54]. Mice in this study were on a C57BL/6 background, obtained by breeding the *Sandy* mice on DBA/2J background with C57BL/6 mice for at least eight generations. Both C57BL/6 and *Sandy* mice were used for primary cortical neuron culture and brain lysate preparation. Pregnant C57BL/6 mice were purchased from Hyochang Science (Daegu, Republic of Korea). All animal procedures were approved by the Institutional Animal Care and Use Committee (IACUC) of Pohang University of Science and Technology (POSTECH-2019-0024). All experiments were carried out in accordance with the approved guidelines.

### Cell culture and transfection

HEK293 cells were cultured in DMEM (HyClone) supplemented with 10% (v/v) fetal bovine serum (FBS) (Gibco) and 1% penicillin/streptomycin (Gibco). All cell lines were authenticated using STR profiling method and were tested negative for mycoplasma contamination. All cells were transfected by using transfection reagent either VivaMagic (Vivagen) or Lipofectamine 2000 (Thermo Fisher Scientific) according to the manufacturer’s instructions.

Primary cultures of cortical neurons were established by isolating E15 mouse embryo cortical tissues in HBSS (Gibco) and dissociating tissues in 0.25% trypsin (Sigma-Aldrich) and 0.1% DNase I (Sigma-Aldrich) for 10 min at 37 °C. Cells were resuspended in neurobasal medium (Gibco) supplemented with 10 mM HEPES pH 7.4 and 10% (v/v) horse serum for final cell concentration being 3.0 × 10^5^ cells/mL and plated on glass coverslips pre-coated with poly-d-lysine and laminin. After 2 h of plating, cell medium was replaced to neurobasal medium containing 2 mM glutamine, 2% (v/v) B27 supplement (Gibco), and 1% (v/v) penicillin/streptomycin. The neurons were transfected at days in vitro (DIV) 7–9 with Lipofectamine 2000 and the medium was replaced with the culture medium 4 h after transfection.

### Antibodies and plasmids

Anti-dysbindin rabbit polyclonal antibody was a kind gift from Dr. Koh-ichi Nagata (Institute for Developmental Research, Japan) and was used as previously described [38, 55]. Anti-p150^glued^ mouse monoclonal antibody (Cat# 612709, BD bioscience), anti-dynein IC1/2, cytosolic mouse monoclonal (Cat# sc-13524, Santa Cruz Biotechnology), anti-mitofilin rabbit polyclonal (Cat# NB100-1919, Novus Biologicals), anti-FLAG rabbit polyclonal and mouse monoclonal (Cat# F7425, and Cat# F1804, respectively, Sigma-Aldrich), anti-α-tubulin mouse monoclonal (Cat# 66031-1-Ig, Proteintech Group), anti-GAPDH mouse monoclonal (Cat# sc-32233, Santa Cruz Biotechnology), anti-GFP rabbit polyclonal (Cat# A-11122, Molecular Probes), and anti-GFP mouse monoclonal (Cat# sc-9996, Santa Cruz Biotechnology) antibodies were used for immunoblotting, IP, and immunostaining experiments. For immunoblotting, HRP-conjugated sheep anti-mouse IgG (Cat# NA931, GE Healthcare) and donkey anti-rabbit IgG (Cat# NA934, GE Healthcare) were used as secondary antibodies. VeriBlot for IP Detection Reagent (HRP) (Cat# ab131366, Abcam) was also used for immunoblotting of IP. For immunostaining, Alexa Fluor 568 conjugated goat anti-rabbit IgG (Cat# A-11011, Molecular Probes), Alexa Fluor 488 conjugated goat anti-mouse IgG (Cat# A-11001, Molecular Probes), 647 conjugated goat anti-rabbit antibodies (Cat# A-21245, Molecular Probes), and Alexa Fluor 405 conjugated goat anti-mouse IgG (Cat# A-31553, Molecular Probes) were used as secondary antibodies.

Constructs for human dysbindin (isoform a, hDysbindin) were prepared by cloning into pFLAG-CMV2 (Sigma-Aldrich) and pEGFP-N1 (Clontech), and constructs for mouse dysbindin (mDysbindin) were prepared by cloning into pFLAG-CMV2, as previously described [38]. To construct the deletion mutants of dysbindin, the regions of human dysbindin and mouse dysbindin corresponding to the designated codons were amplified by PCR using GFP-hDysbindin or shRNA-resistant form of FLAG-mDysbindin as a template and inserted into the pEGFP-N1 or pFLAG-CMV2, respectively, as previously described [38]. Constructs for human p150^glued^ (dynactin subunit 1 isoform 1) were cloned into pFLAG-CMV2 and pEGFP-N1, human dynamitin (dynactin subunit 2 isoform 1) was cloned into pFLAG-CMV2, and human dynein intermediate chain (dynein IC) was cloned into pEGFP-C3 (Clontech). Mitochondrial transit sequence (MTS)-mCherry was prepared as previously described [53], pGP-CMV-GCaMP6s was a gift from Douglas Kim and GENIE Project (Cat# 40753) [56], and pCAG-vGlut1-GCaMP5G was a kind gift from Dr. Seok-Kyu Kwon (Korea Institute of Science and Technology, Republic of Korea). The oligonucleotide sequences used for mouse dysbindin shRNA were GTGATAAGTCAAGAGAAGCTTCAAGAGAGCTTCTCTTGACTTACACTTTTTT, TCGAGAAAAAAGTGATAAGTCAAGAGAAGCTCTCTTGAAGCTTCTCTTGACTTATCACA, as previously described [38], and the sequence for human dysbindin shRNA was AAGTGACAAGTCAAGAGAAGC. The oligonucleotide sequences for mouse p150^glued^ shRNA were GACTTCACCCCTTGATTAA and CGAGCTCACCACAGACCTG [57]. These oligonucleotides were annealed and ligated into the pLentiLox3.7 vector using PstI and XhoI sites. The shRNA-resistant FLAG-mDysbindin construct was generated by site-directed mutagenesis, and the sequence for the PCR primer was AAGACTTTA AGTGACAAATCAAGGGAGGCAAAAGTGAAA as previously described [38].

### Immunoprecipitation and immunoblotting

Transfected HEK293 cells were lysed in 1× ELB lysis buffer [50 mM Tris, pH 8.0, 250 mM NaCl, 0.1% NP-40, 5 mM EDTA, 2 mM NaPPi, 10 mM NaF, 2 mM Na_3_VO_4_, 1 mM DTT, and protease inhibitor cocktail (Roche)] and mouse brain tissues were homogenized and lysed in 1× NP40 lysis buffer (50 mM Tris, pH 8.0, 150 mM NaCl, 1% NP-40, 5 mM EDTA, 2 mM NaPPi, 10 mM NaF, 2 mM Na_3_VO_4_, 1 mM DTT, and protease inhibitor cocktail).

For immunoprecipitation, the lysates were incubated with 1–2 µg of antibody at 4 °C overnight on a rocking platform. Protein-A agarose beads (Roche) resuspended in the lysis buffer were mixed with immunoprecipitated lysates and incubated for 2–3 h at 4 °C with constant rotation. The precipitates were washed three times with the lysis buffer and mixed with SDS sample buffer for immunoblotting.

For immunoblotting, proteins were denatured by mixing lysates with 5× SDS sample buffer (2% SDS, 60 mM Tris pH 6.8, 24% glycerol, and 0.1% bromophenol blue, and 5% β-mercaptoethanol) and boiling at 100 °C for 6 min. Proteins were separated by SDS-PAGE with 8.5% polyacrylamide gel and transferred to PVDF membrane (Millipore). Membranes were blocked with 5% skim milk in Tris-buffered saline (20 mM Tris pH 8.0, and 137.5 mM NaCl) with 0.25% Tween20 (TBST) for 1 h and incubated with primary antibodies at 4 °C overnight and HRP-conjugated secondary antibodies at room temperature for 2 h. Protein signals were detected by ECL solutions (BioRad).

### Immunocytochemistry

Primary cultured cortical neurons at DIV 10 were fixed with 4% paraformaldehyde and 4% sucrose in PBS for 15 min and permeabilized with 0.2% Triton X-100 in PBS for 5 min and incubated in the blocking solution (3% BSA in PBS) for 30 min. Neurons were incubated with rabbit anti-FLAG antibody, mouse anti-GFP antibody, or mouse anti-α-tubulin antibody diluted in the blocking solution for 2 h at room temperature or overnight at 4 °C, washed with PBS for three times, followed by incubation with Alexa Fluor 568-conjugated secondary antibody, Alexa Fluor 488-conjugated secondary antibody, Alexa Fluor 647-conjugated secondary antibody, or Alexa Fluor 405-conjugated secondary antibody for 2 h at room temperature.

Images were obtained using FV3000 confocal laser scanning microscope (Olympus) with UPLSAPO 40×/0.95 NA objective or UPLSAPO 100×/1.45 NA oil objective and deconvolved using advanced constrained iterative (CI) algorithm-based deconvolution program of cellSens (Olympus).

### Mitochondrial transport imaging and analysis

Mitochondrial transport imaging was performed following the previous description with modifications [53, 58]. Primary cultured cortical neurons at DIV 7–9 were transfected with MTS-mCherry and indicated constructs. At DIV 10–12, live time-lapse imaging was performed using FV3000 confocal laser scanning microscope (Olympus) with UPLSAPO 20×/0.75 NA objective at 37 °C with supplying 5% CO_2_ gas. Neurons were imaged for 2 min with 3 s interval. The obtained images were subjected to the analyses for the mitochondrial motility using cellSens (Olympus). Based on the morphological criteria, an axon was identified as a long and thin process, while dendrites were shorter and thicker. In axons, a 150 µm segment from at least 100 μm away from soma was selected as a proximal axon, and a 150 µm segment from the axon tip was selected as a distal axon. For the analysis of dendrites, a 100 µm segment of the longest dendrite 100 µm away from soma was analyzed. Mitochondrion showing a displacement from the original point at least 5 μm for 2 min was regarded as a motile one. The motile and stationary mitochondria were counted manually based on the image sequences and kymographs generated by cellSens (Olympus). The motility of mitochondria was presented as the percentage of motile mitochondria.

### Calcium imaging and analysis

Live calcium imaging was performed following previous descriptions with modifications [59, 60]. Primary cultured cortical neurons at DIV 7–9 were transfected with cyto-GCaMP6s or vGlut1-GCaMP5G and indicated constructs followed by time-lapse calcium imaging at DIV 10–12. Primary cultured neurons were loaded with low potassium buffer (10 mM HEPES, pH 7.4, 126 mM NaCl, 4 mM KCl, 2 mM CaCl_2_, 1 mM MgCl_2_, 4.2 mM glucose) and treated with 50 mM KCl. The fluorescence intensities were recorded in FV31S-DT software at an interval of 2 s for total 180 s for cyto-GCaMP6s and total 120 s for vGlut1-GCaMP5G using FV3000 confocal laser scanning microscope (Olympus) with UPLSAPO 20×/0.75 NA objective at 37 °C with supplying 5% CO_2_ gas. Background fluorescence was subtracted and the amplitude (∆F/F_0_) of each neuron was calculated as (F−F_0_)/F_0_, where F_0_ is a baseline GCaMP fluorescence signal averaged over 30 s before the stimulation and F is a peak intensity of fluorescence in the response. The trace of calcium decay of each neuron was fit with nonlinear regression using GraphPad Prism. The decay time to half-maximum was obtained by the half-life of the monoexponential curve.

### Microtubule co-sedimentation assay

Microtubule co-sedimentation assay was carried out as a previous description with modifications [53, 61]. Briefly, brains isolated from WT and *Sandy* mice were homogenized in microtubule stabilizing buffer (100 mM PIPES (pH 6.8), 0.1% NP40, 5 mM MgCl_2_, 2 mM EGTA, 100 mM NaF, 2 M Glycerol, and protease inhibitor cocktail) and centrifuged at 20,000×*g* for 40 min at 4 °C. The supernatant was treated with 30 μM Taxol and incubated for 30 min at 37 °C, followed by centrifugation at 12,000×*g* for 40 min at room temperature. The pellets were resuspended in microtubule stabilizing buffer and supernatant was dissolved in the SDS-PAGE sample buffer and subjected to the western blot analysis.

### Mitochondrial fractionation

Mitochondrial fractionation from primary cultured neurons was performed as a previous description with modifications [53]. Additionally, AraC (#Cat C1768, Sigma-Aldrich) was added to the neurons at DIV 3 for 24 h to inhibit the growth of non-neuronal cells. Harvested neurons at DIV 11 were washed with PBS, suspended in mitochondria isolation buffer (250 mM sucrose, 1 mM EGTA, 1 mM MgCl_2_, 0.5 mM DTT, 10 mM Tris, pH 8.0), and disrupted by dounce homogenization. The homogenate was spun at 800×*g* for 10 min at 4 °C twice. The supernatant was recovered and centrifuged again at 8000×*g* for 10 min at 4 °C. The resulting pellet (mitochondrial fraction) was collected, and the supernatant (cytosolic fraction) was cleared by further centrifugation at 12,000×*g* for 10 min at 4 °C.

Mitochondrial fractionation from mouse brain lysates was performed as previously described [62]. A brain was rinsed and homogenized in isolation buffer-1 (225 mM mannitol, 75 mM sucrose, 0.5% BSA, 0.5 mM EGTA, 30 mM Tris–HCl pH 7.4) and centrifuged at 740×*g* for 5 min at 4 °C. A small portion of the supernatant was kept as a whole lysate fraction and the rest was centrifuged at 9000×*g* for 10 min at 4 °C. The supernatant was kept as a cytosolic fraction, the pellet was resuspended in isolation buffer-2 (225 mM mannitol, 75 mM sucrose, 0.5% BSA, 30 mM Tris–HCl pH 7.4) and centrifuge at 10,000×*g* for 10 min at 4 °C. The resultant pellet was resuspended in isolation buffer-3 (225 mM mannitol, 75 mM sucrose, 30 mM Tris–HCl pH 7.4) and centrifuged at 10,000×*g* for 10 min at 4 °C. The pellet was resuspended in MRB (mitochondria resuspending buffer) (250 mM mannitol, 5 mM HEPES (pH 7.4), 0.5 mM EGTA) and a small portion was collected as crude mitochondria fraction. This fraction was loaded on percoll medium (225 mM mannitol, 25 mM HEPES (pH 7.4), 1 mM EGTA, 30% Percoll (vol/vol)) and centrifuged at 95,000×*g* for 30 min at 4 °C in a Beckman Coulter Optima XE-100 Ultracentrifuge (SW41 Ti rotor). The lower bands were collected and diluted with MRB and centrifuged at 6,300×*g* for 10 min at 4 °C. The pellet was collected as pure mitochondria fraction and resuspended in MRB.

### Statistical analysis

All graphs were analyzed using the GraphPad Prism software and presented as the mean ± standard error of mean (SEM). Statistical significance of the data was determined by two-tailed Student’s *t* test for comparisons between two groups and one-way analysis of variance (ANOVA) followed by Bonferroni’s post-hoc test for comparisons among multiple groups (Additional files 5, 6).

## Supplementary Information

**Additional file 1: Fig. S1.** Altered mitochondrial movement and density in axons upon dysbindin knockdown and overexpression. **a** Quantitative analyses of mitochondrial movement and **b** anterograde and retrograde movements in the proximal and distal axons of DIV 10–12 primary cortical neurons transfected as indicated. **c** Quantification of mitochondrial density (the number of mitochondria per 100 µm) in the proximal and distal axons of DIV 10–12 primary cortical neurons (n = 23 axons for CTL shRNA, n = 33 axons for Dysbindin shRNA, and n = 24 axons for Dysbindin shRNA + mDysbindin for the proximal axons, n = 14 axons for CTL shRNA, n = 14 axons for Dysbindin shRNA, and n = 7 axons for Dysbindin shRNA + mDysbindin for the distal axons). **d** Quantitative analyses of mitochondrial movement, and **e** anterograde and retrograde movements in the proximal and distal axons of DIV 10–12 primary cortical neurons transfected with FLAG-mDysbindin. **f** Quantification of mitochondrial density in the proximal and distal axons of DIV 10–12 primary cortical neurons (n = 38 axons for CTL, and n = 37 axons for mDysbindin for the proximal axons, n = 18 axons for CTL, and n = 21 axons for mDysbindin for the distal axons). **g** Quantitative analysis of mitochondrial movement and **h** mitochondrial density in dendrites of DIV 10–12 primary cortical neurons transfected as indicated (n = 34 dendrites for CTL shRNA, n = 27 dendrites for Dysbindin shRNA). All results are presented as the mean $\pm$ SEM. *p < 0.05, **p < 0.01, and ***p < 0.001 from one-way ANOVA with Bonferroni’s multiple comparison test for a, b, and c, and Student’s *t*-test for d, e, f, g, and h.

**Additional file 2: Fig. S2.** Detection of endogenous dysbindin and p150^glued^ from the mitochondrial fraction and reduced interaction of p150^glued^-dynamitin upon dysbindin knockdown. **a** Endogenous dysbindin was detected from the mitochondrial fraction of mouse brain lysates by western blotting with anti-dysbindin antibody. Mitofilin and GAPDH were used as markers for the mitochondrial and cytosolic fractions, respectively. **b** The protein level of endogenous p150^glued^ was compared between the mitochondrial fractionation of WT and *Sandy* mouse brain lysates. Endogenous p150^glued^ was detected by western blotting with anti-p150^glued^ antibody. Mitofilin and α-tubulin were used as markers for the mitochondrial and cytosolic fractions, respectively. **c** Knockdown of endogenous dysbindin by human dysbindin shRNA in HEK293 cells. **d** Quantification of the protein level of dysbindin normalized by GAPDH. **e** Co-immunoprecipitation of p150^glued^ and dynamitin upon dysbindin knockdown. Lysates from HEK293 cells transfected with the indicated shRNA were immunoprecipitated with anti-p150^glued^ antibody. Immunoprecipitates were analyzed by western blotting with anti-dynamitin and anti-p150^glued^. **f** Quantification of the protein level of co-immunoprecipitated dynamitin normalized by immunoprecipitated p150^glued^. Asterisks indicate the protein of interest. All results are presented as the mean $\pm$ SEM. *p < 0.05, **p < 0.01, and ***p < 0.001 from Student’s *t*-test.

**Additional file 3:** Fig. S3. Mitochondrial density in the proximal axon and mitochondrial motility in the distal axon affected by p150^glued^ and dysbindin. **a** Quantification of mitochondrial density in the proximal axons of DIV 10–12 primary cortical neurons transfected as indicated (n = 32 axons for CTL shRNA, n = 33 axons for p150^glued^ shRNA, n = 14 axons for Dysbindin shRNA, and n = 24 axons for p150^glued^ shRNA + Dysbindin shRNA). **b** Quantitative analysis of mitochondrial movement in the distal axons of DIV 10–12 primary cortical neurons (n = 26 axons for CTL shRNA, n = 12 axons for p150^glued^ shRNA, n = 9 axons for Dysbindin shRNA, and n = 17 axons for p150^glued^ shRNA + Dysbindin shRNA). **c** Quantification of mitochondrial density in the proximal axons of DIV 10–12 primary cortical neurons transfected as indicated (n = 34 axons for CTL shRNA, n = 30 axons for p150^glued^ shRNA, n = 32 axons for mDysbindin, and n = 33 axons for p150^glued^ shRNA + mDysbindin). **d** Quantitative analysis of mitochondrial movement in the distal axons of DIV 10–12 primary cortical neurons (n = 49 axons for CTL shRNA, n = 27 axons for p150^glued^ shRNA, n = 34 axons for mDysbindin, and n = 30 axons for p150^glued^ shRNA + mDysbindin). All results are presented as the mean $\pm$ SEM. *p < 0.05, **p < 0.01, and ***p < 0.001 from one-way ANOVA with Bonferroni’s multiple comparison test.

**Additional file 4: Fig. S4.** Calcium dynamics in presynaptic terminals of *Sandy* neurons and presynaptic boutons of dysbindin knockdown neurons. Live-cell calcium imaging for monitoring calcium dynamics. **a** Calcium response graph obtained after 50 mM KCl stimulation in DIV 10–12 primary cortical neurons expressing cyto-GCaMP6 of WT and *Sandy* mice. **b** Statistically analyzed peak amplitudes, **c** the area under the curves, and **d** the decay time to half-maximum of the calcium response graphs (n = 45 for WT and n = 51 for *Sandy*). **e** Calcium response graph obtained after 50 mM KCl stimulation in DIV 10–12 primary cortical neurons expressing vGlut1-GCaMP5 and the indicated shRNA. **f** Statistically analyzed peak amplitudes and **g** the area under the curves (n = 92 for CTL shRNA and n = 134 for Dysbindin shRNA). All results are presented as the mean $\pm$ SEM. *p < 0.05, **p < 0.01, and ***p < 0.001 from Student’s *t*-test.

**Additional file 5:** Raw data - quantified  numerical data.

**Additional file 6:** Raw data - raw images of western blotting.

## Data Availability

The data generated or analyzed during this study are included in this published article and its supplementary information files.

## Abbreviations

Aβ: Amyloid β
ALS: Amyotrophic lateral sclerosis
ANOVA: Analysis of variance
AP-3: Adaptor related protein complex-3
BLOC-1: Biogenesis of lysosome-related organelle complex 1
CAP-Gly: Cytoskeleton-associated protein glycine-rich
CTL: Control
DISC1: Disrupted-in-schizophrenia 1
DIV: Days in vitro
IC: Intermediate chain
EGFP: Enhanced green fluorescent protein
EM: Electron microscopy
ER: Endoplasmic reticulum
GABA: Gamma-aminobutyric acid
GFP: Green fluorescent protein
HEK: Human embryonic kidney
IP: Immunoprecipitation
KCl: Potassium chloride
MTS: Mitochondrial transit sequence
SBMA: Spinobulbar muscular atrophy
SEM: Standard error of mean
shRNA: Short hairpin ribonucleic acid
WT: Wild-type
