# Supplementary figures and images for: Schizophrenia-associated dysbindin modulates axonal mitochondrial movement in cooperation with p150glued
Source: Mol Brain. 2021 Jan 18;14:14. doi: 10.1186/s13041-020-00720-3 (PMC7814725; doi:10.1186/s13041-020-00720-3)

Additional file 1: Fig. S1

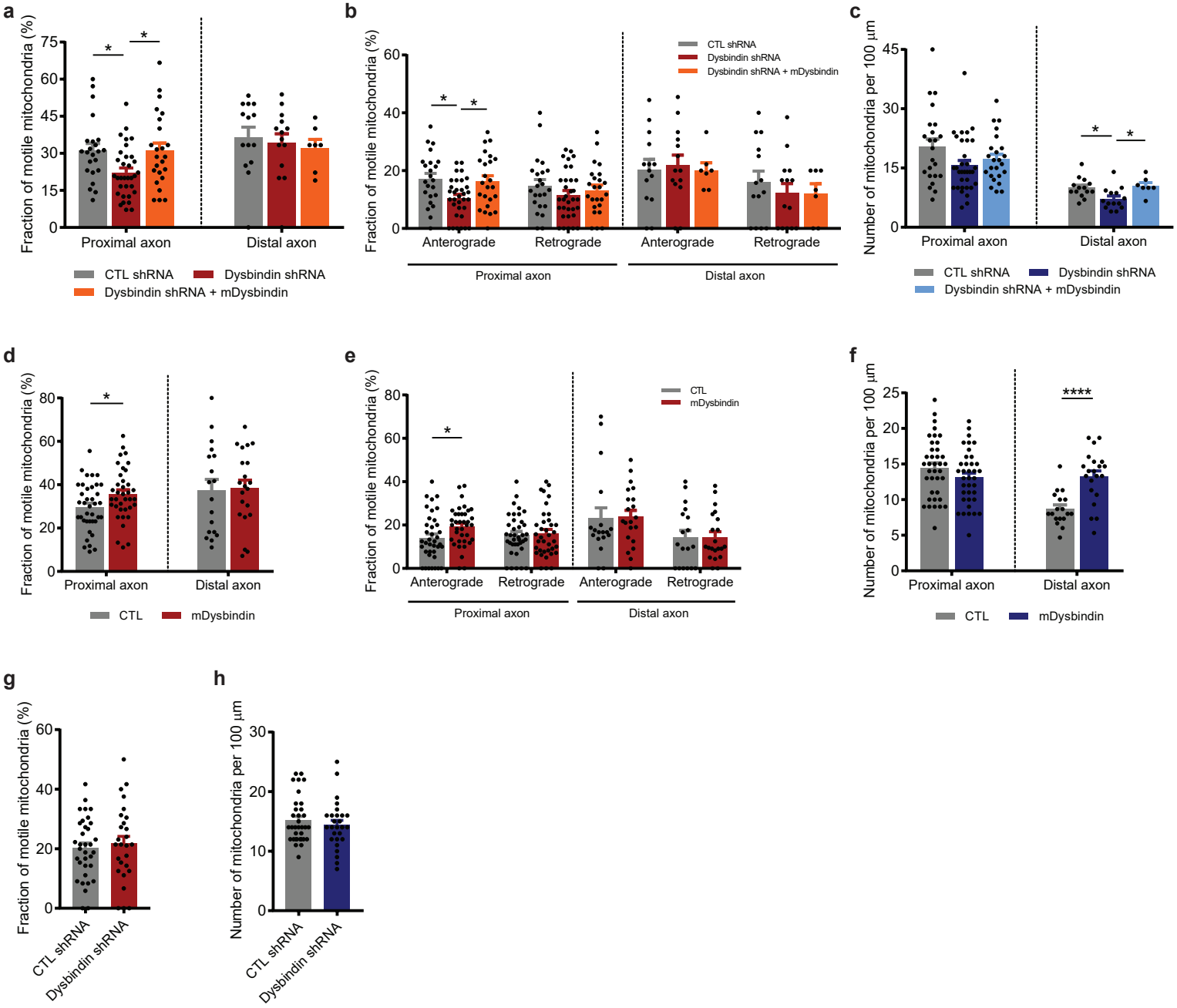

Supplement: Supplementary file 1 — Additional file 1: Fig. S1. Altered mitochondrial movement and density in axons upon dysbindin knockdown and overexpression. a Quantitative analyses of mitochondrial movement and b anterograde and retrograde movements in the proximal and distal axons of DIV 10–12 primary cortical neurons transfected as indicated. c Quantification of mitochondrial density (the number of mitochondria per 100 µm) in the proximal and distal axons of DIV 10–12 primary cortical neurons (n = 23 axons for CTL shRNA, n = 33 axons for Dysbindin shRNA, and n = 24 axons for Dysbindin shRNA + mDysbindin for the proximal axons, n = 14 axons for CTL shRNA, n = 14 axons for Dysbindin shRNA, and n = 7 axons for Dysbindin shRNA + mDysbindin for the distal axons). d Quantitative analyses of mitochondrial movement, and e anterograde and retrograde movements in the proximal and distal axons of DIV 10–12 primary cortical neurons transfected with FLAG-mDysbindin. f Quantification of mitochondrial density in the proximal and distal axons of DIV 10–12 primary cortical neurons (n = 38 axons for CTL, and n = 37 axons for mDysbindin for the proximal axons, n = 18 axons for CTL, and n = 21 axons for mDysbindin for the distal axons). g Quantitative analysis of mitochondrial movement and h mitochondrial density in dendrites of DIV 10–12 primary cortical neurons transfected as indicated (n = 34 dendrites for CTL shRNA, n = 27 dendrites for Dysbindin shRNA). All results are presented as the mean $\pm$± SEM. *p < 0.05, **p < 0.01, and ***p < 0.001 from one-way ANOVA with Bonferroni’s multiple comparison test for a, b, and c, and Student’s t-test for d, e, f, g, and h. [file 13041_2020_720_MOESM1_ESM.pdf]

Additional file 2: Fig. S2

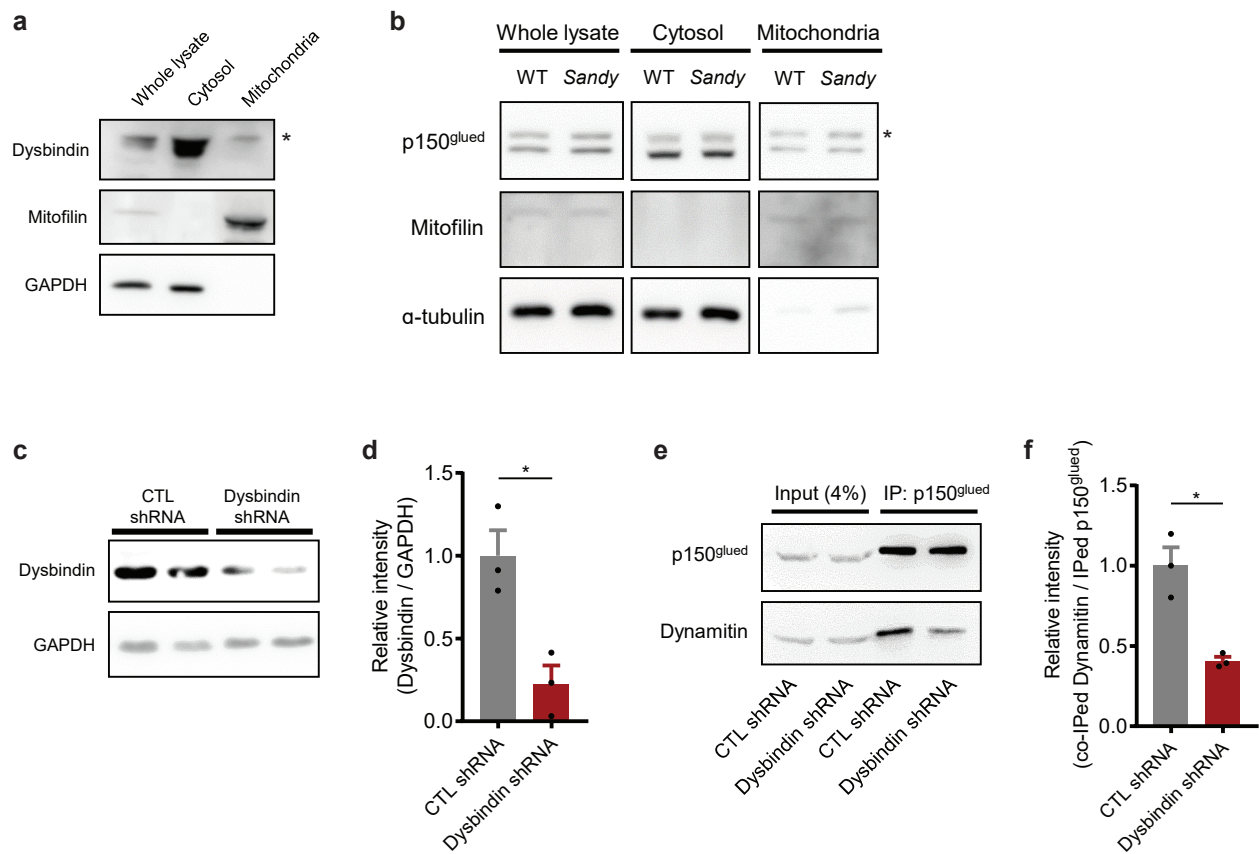

Supplement: Supplementary file 2 — Additional file 2: Fig. S2. Detection of endogenous dysbindin and p150glued from the mitochondrial fraction and reduced interaction of p150glued-dynamitin upon dysbindin knockdown. a Endogenous dysbindin was detected from the mitochondrial fraction of mouse brain lysates by western blotting with anti-dysbindin antibody. Mitofilin and GAPDH were used as markers for the mitochondrial and cytosolic fractions, respectively. b The protein level of endogenous p150glued was compared between the mitochondrial fractionation of WT and Sandy mouse brain lysates. Endogenous p150glued was detected by western blotting with anti-p150glued antibody. Mitofilin and α-tubulin were used as markers for the mitochondrial and cytosolic fractions, respectively. c Knockdown of endogenous dysbindin by human dysbindin shRNA in HEK293 cells. d Quantification of the protein level of dysbindin normalized by GAPDH. e Co-immunoprecipitation of p150glued and dynamitin upon dysbindin knockdown. Lysates from HEK293 cells transfected with the indicated shRNA were immunoprecipitated with anti-p150glued antibody. Immunoprecipitates were analyzed by western blotting with anti-dynamitin and anti-p150glued. f Quantification of the protein level of co-immunoprecipitated dynamitin normalized by immunoprecipitated p150glued. Asterisks indicate the protein of interest. All results are presented as the mean $\pm$± SEM. *p < 0.05, **p < 0.01, and ***p < 0.001 from Student’s t-test. [file 13041_2020_720_MOESM2_ESM.pdf]

Additional file 3: Fig. S3

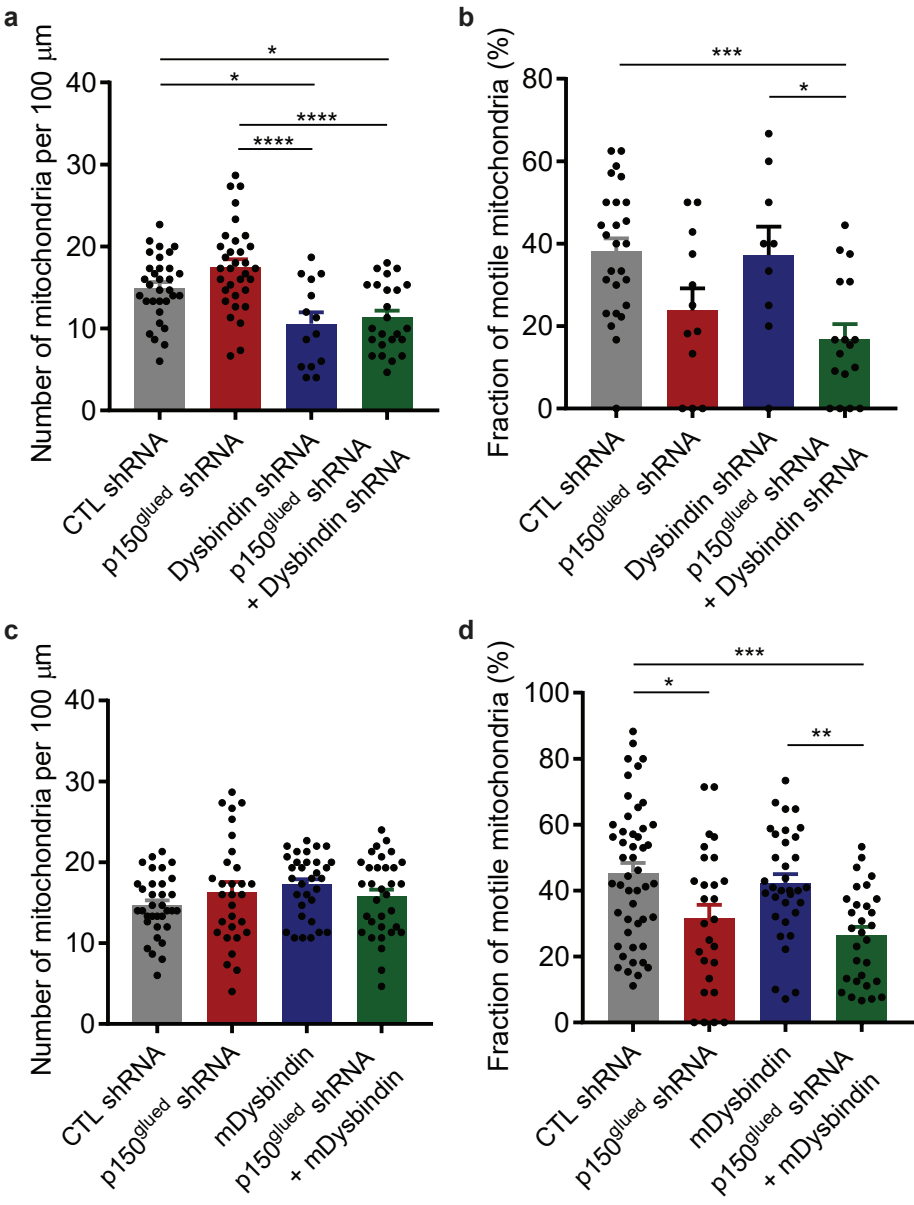

Supplement: Supplementary file 3 — Additional file 3: Fig. S3. Mitochondrial density in the proximal axon and mitochondrial motility in the distal axon affected by p150glued and dysbindin. a Quantification of mitochondrial density in the proximal axons of DIV 10–12 primary cortical neurons transfected as indicated (n = 32 axons for CTL shRNA, n = 33 axons for p150glued shRNA, n = 14 axons for Dysbindin shRNA, and n = 24 axons for p150glued shRNA + Dysbindin shRNA). b Quantitative analysis of mitochondrial movement in the distal axons of DIV 10–12 primary cortical neurons (n = 26 axons for CTL shRNA, n = 12 axons for p150glued shRNA, n = 9 axons for Dysbindin shRNA, and n = 17 axons for p150glued shRNA + Dysbindin shRNA). c Quantification of mitochondrial density in the proximal axons of DIV 10–12 primary cortical neurons transfected as indicated (n = 34 axons for CTL shRNA, n = 30 axons for p150glued shRNA, n = 32 axons for mDysbindin, and n = 33 axons for p150glued shRNA + mDysbindin). d Quantitative analysis of mitochondrial movement in the distal axons of DIV 10–12 primary cortical neurons (n = 49 axons for CTL shRNA, n = 27 axons for p150glued shRNA, n = 34 axons for mDysbindin, and n = 30 axons for p150glued shRNA + mDysbindin). All results are presented as the mean $\pm$± SEM. *p < 0.05, **p < 0.01, and ***p < 0.001 from one-way ANOVA with Bonferroni’s multiple comparison test. [file 13041_2020_720_MOESM3_ESM.pdf]

Additional file 4: Fig. S4

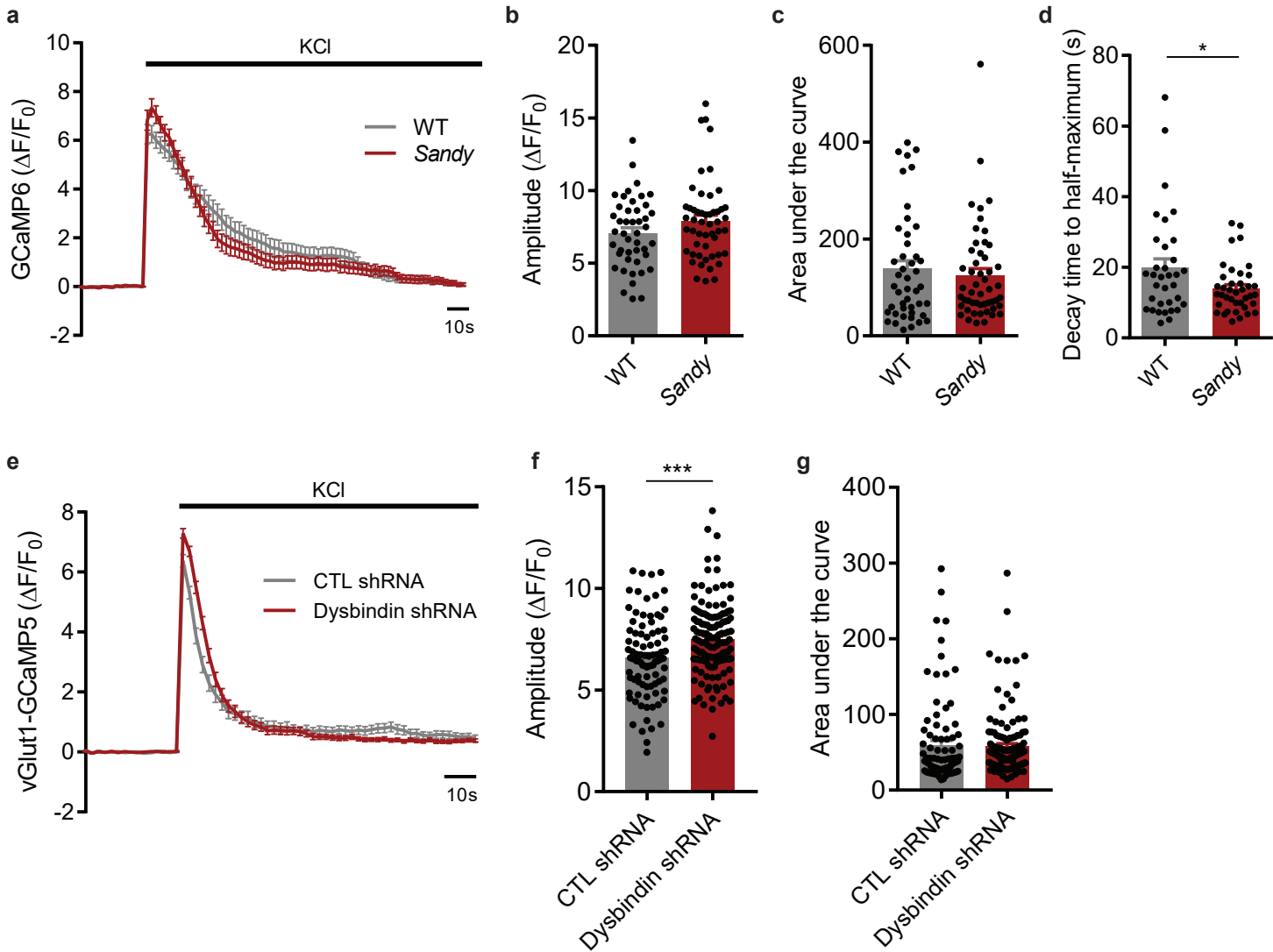

Supplement: Supplementary file 4 — Additional file 4: Fig. S4. Calcium dynamics in presynaptic terminals of Sandy neurons and presynaptic boutons of dysbindin knockdown neurons. Live-cell calcium imaging for monitoring calcium dynamics. a Calcium response graph obtained after 50 mM KCl stimulation in DIV 10–12 primary cortical neurons expressing cyto-GCaMP6 of WT and Sandy mice. b Statistically analyzed peak amplitudes, c the area under the curves, and d the decay time to half-maximum of the calcium response graphs (n = 45 for WT and n = 51 for Sandy). e Calcium response graph obtained after 50 mM KCl stimulation in DIV 10–12 primary cortical neurons expressing vGlut1-GCaMP5 and the indicated shRNA. f Statistically analyzed peak amplitudes and g the area under the curves (n = 92 for CTL shRNA and n = 134 for Dysbindin shRNA). All results are presented as the mean $\pm$± SEM. *p < 0.05, **p < 0.01, and ***p < 0.001 from Student’s t-test. [file 13041_2020_720_MOESM4_ESM.pdf]
