# Supplementary material for: Schizophrenia-associated dysbindin modulates axonal mitochondrial movement in cooperation with p150glued
Source: Mol Brain. 2021 Jan 18;14:14. doi: 10.1186/s13041-020-00720-3 (PMC7814725; doi:10.1186/s13041-020-00720-3)

Raw images of western blotting

(Lanes correspond to quantified data are marked in blue)

Figure 2a

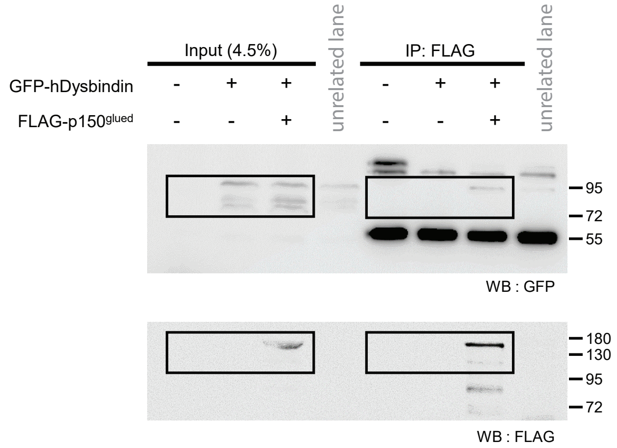

Figure 2c

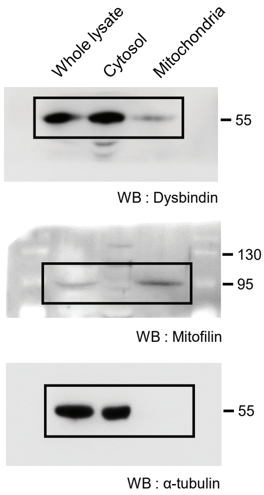

Figure 2e-g - quantification of p150^{glued} / α-tubulin , Dynein IC / α-tubulin

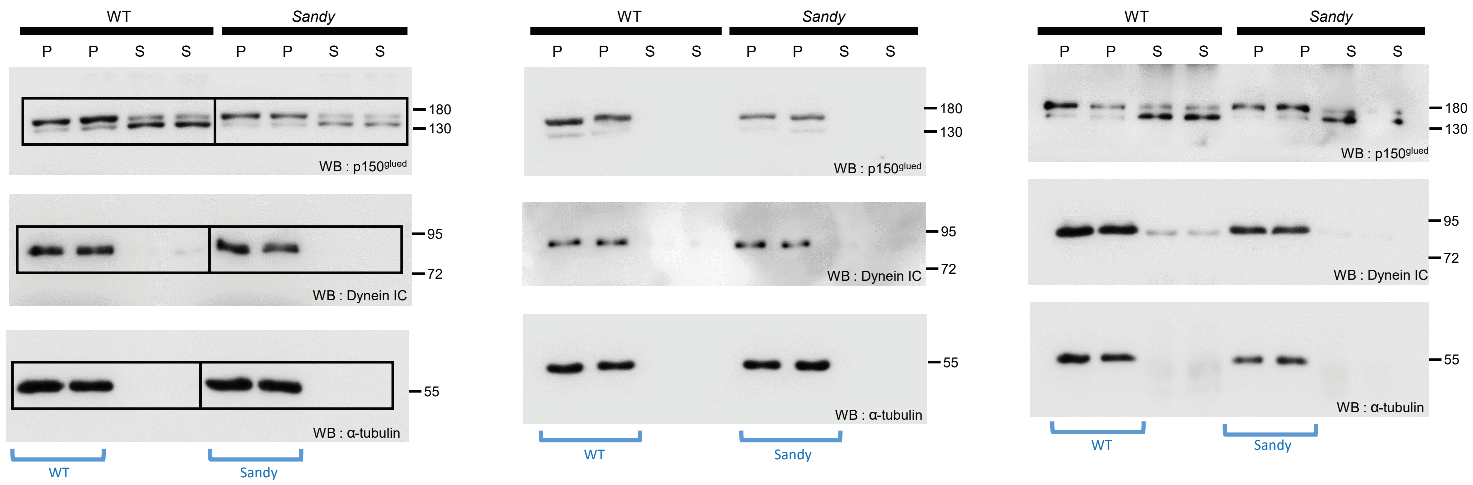

Figure 2h, i - quantification of co-IPed p150^{glued} / IPed Dynein IC

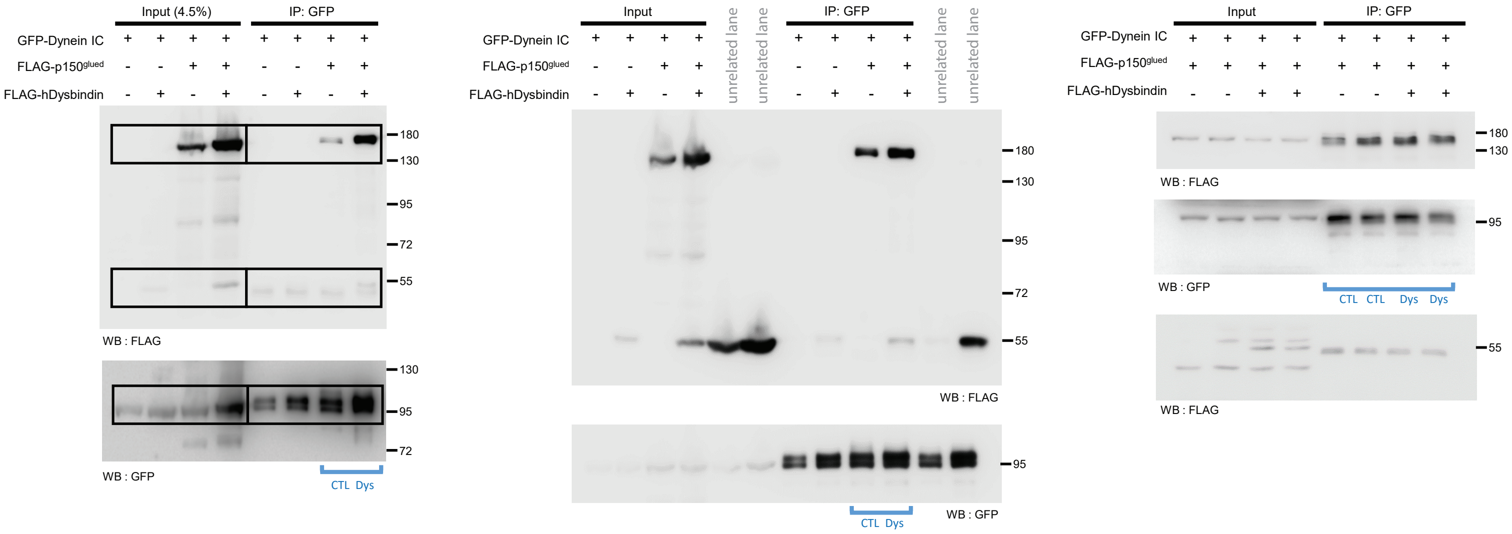

Figure 2j, k - quantification of co-IPed p150^{glued} / IPed Dynamitin

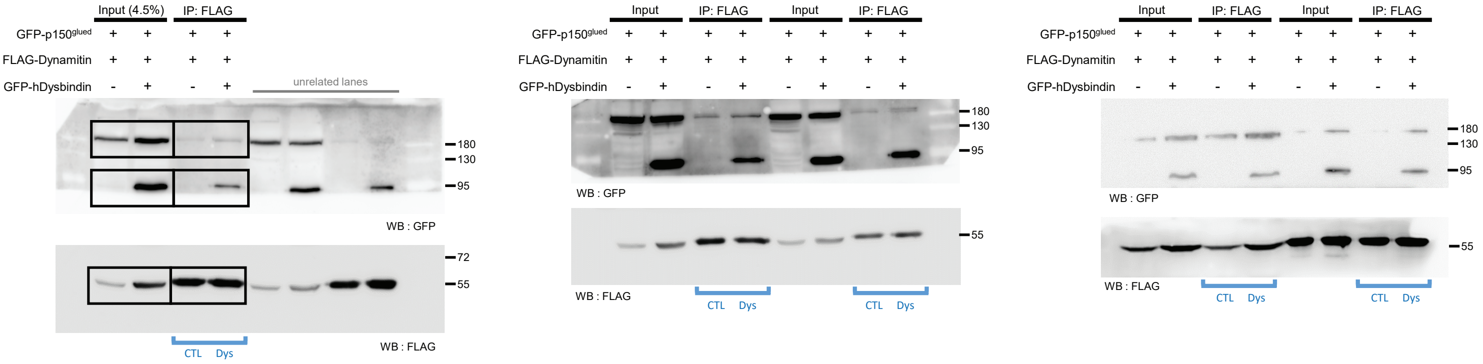

Figure 3h

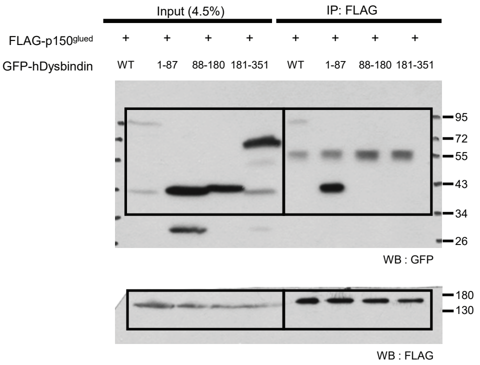

Figure S2a

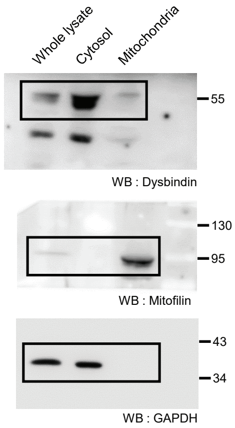

Figure S2b

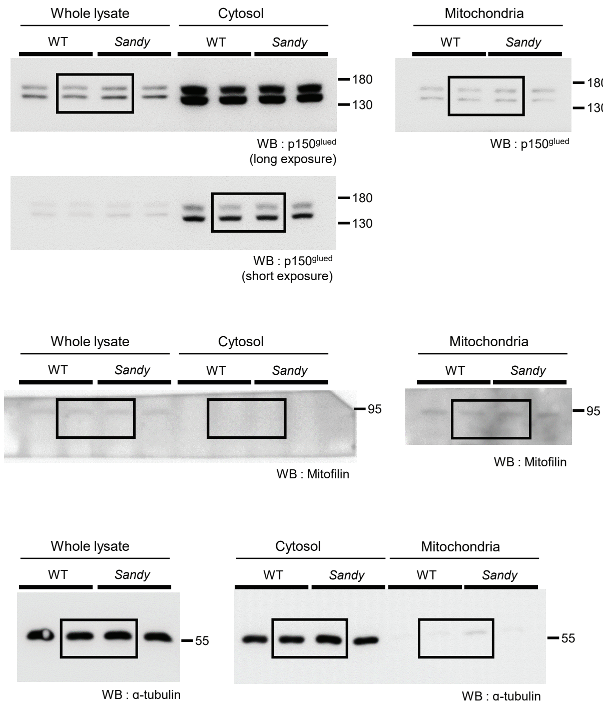

Figure S2c, d - quantification of Dysbindin / GAPDH

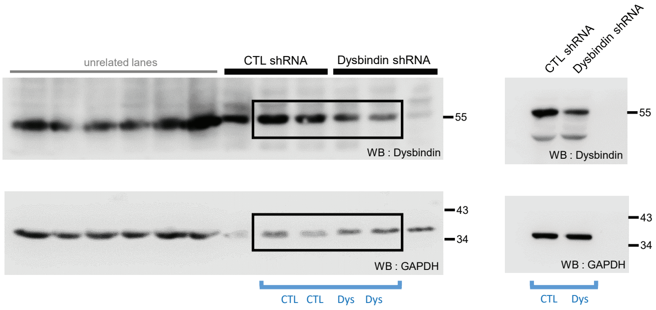

Figure S2e, f - quantification of co-IPed Dynamitin / IPed p150^{glued}

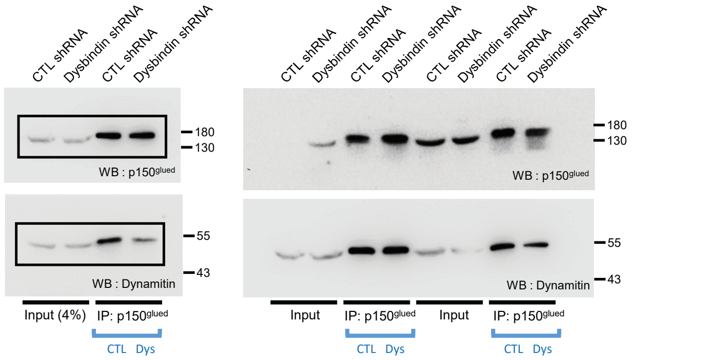

Supplement: Supplementary file 6 — Additional file 6: Raw data - raw images of western blotting. [file 13041_2020_720_MOESM6_ESM.pdf]
